# The Rise of Plant‐Based Proteins: Consumer Perception and Challenges

**DOI:** 10.1002/fsn3.71713

**Published:** 2026-04-29

**Authors:** Batuhan Inanlar, Esra Capanoglu

**Affiliations:** ^1^ Faculty of Chemical & Metallurgical Engineering, Department of Food Engineering Istanbul Technical University Istanbul Türkiye

## Abstract

Proteins are vital for human health, and with the rise in population and environmental problems, plant‐based proteins are becoming more popular. They have many benefits, such as preventing several chronic diseases and contributing to sustainability by decreasing greenhouse gas emissions, land use, and water consumption. Furthermore, the plant‑based food market in the EU was valued at approximately €9 billion in 2024, with milk, beverages, and meat alternatives accounting for a large share of total sales. Plant‐based proteins account for 80% of global protein consumption and come from various sources, making them a more sustainable alternative to animal proteins. However, they also have several limitations, including the lack of some essential amino acids and lower bioavailability compared to animal proteins. There are also several challenges, such as negative perceptions of taste and regulatory restrictions on product labeling. Barriers such as food neophobia, taste, and accessibility affect their consumption. Regulations, new production methods, and more research can improve consumer adoption of plant‐based proteins, which could position them as the food of the future due to their health and ecological advantages. This paper discusses the benefits and challenges of plant‐based foods, various plant protein sources, and research on consumer perceptions of these proteins.

## Graphical abstract

The health, economic, and environmental impacts of plant proteins, research, R&D investments, and regulations have driven the plant‐based food market to rise. Considering these developments and the challenges related to plant‐based foods, we have compiled research on plant‐based proteins from the perspective of consumers in various countries. From a consumer perspective, while different consumers evaluate plant‐based proteins and alternatives similarly, there is a general lack of information, and issues such as price, taste, and accessibility influence consumer choices.

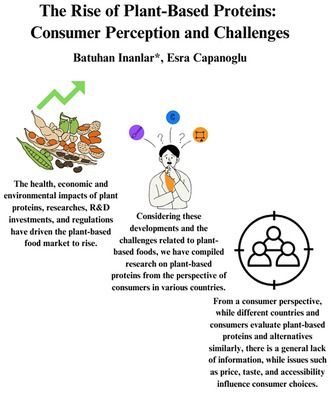

## 1 Introduction

Proteins are macromolecules made up of amino acids that support the growth and maintenance of cells and tissues. Protein requirements vary across life stages. The European Food Safety Authority (EFSA) advises adults to consume at least 0.83 g of protein per kg of body weight per day. Plant and animal proteins differ in their properties and digestibility. However, this may be less critical when total protein intake is adequate (EUFIC 2019). There are 20 standard amino acids. The body synthesizes some amino acids, whereas others must be obtained from the diet. Those that the body cannot produce are defined as “essential amino acids,” which must be obtained from food. Nine of the 20 amino acids are essential, including histidine, leucine, and lysine. On the other hand, “non‐essential amino acids” can be produced by the body and are not required from the diet. Eleven amino acids are non‐essential, including alanine, glutamine, and serine. The number and sequence of amino acids determine a protein's structure and functional specificity. The human body produces over 1000 proteins, each with distinct biological functions. Proteins help build and repair tissues, produce hormones, and support brain function. They also provide energy at approximately 4 kcal/g. Additionally, proteins are essential for maintaining hair and skin health and supporting muscle growth (Cleveland Clinic 2021).

Protein deficiency compromises these functions, and mood changes may result due to their role in neurotransmitter synthesis. Other symptoms of protein deficiency include hunger, fatigue, and blood sugar fluctuations (UCLA Health 2020). It is estimated that nearly a billion people suffer from protein deficiency. The most affected regions are Central Africa and South Asia, where nearly one‐third of children are protein deficient (Alliance for Science 2020). By 2050, the world population is expected to reach approximately 9.7 billion. To feed this growing population, global food production will need to increase by approximately 70%. However, the current food system is relatively low in diversity, with only 12 plant and five animal species providing 75% of the global food supply (Colgrave et al. 2021). A 1°C–2°C increase in global temperature is expected to reduce crop yields and biodiversity globally (United Nations 2016). Plant‐based foods can help mitigate climate change due to their lower greenhouse gas emissions and also provide numerous health benefits (United Nations 2021).

This review explores the benefits and challenges of plant‐based proteins, highlighting their significance in both human health and environmental sustainability. It also examines consumer perceptions to provide insights into the role of plant‐based foods in modern diets. These insights may inform future strategies to improve product quality, address existing barriers, and align offerings with consumer expectations.

## 2 Importance of Plant‐Based Foods

The World Health Organization recommends plant‐based foods due to their health and environmental benefits. These foods not only improve human health but also help protect the environment (WHO 2021). In this section, the importance of plant‐based foods is examined from economic, sustainability‐related, and health perspectives.

### 2.1 Economic Benefits of Plant‐Based Foods Market

The global economy is transitioning toward a circular model to establish a sustainable system in which waste is minimized and resources are preserved. Plant‐based products play a significant role in this circular economy by shortening supply chains and increasing the turnover of products. Their greater recyclability also provides an advantage in terms of raw material efficiency (Shogren et al. 2019).

Plant‐based foods are gaining popularity. According to Good Food Institute (GFI) Europe, Europe’s plant‑based retail market was valued at approximately €9 billion in 2024, with plant‑based products including milk and drinks and meat accounting for a large share of total sales across key markets like France, Germany, Italy, Spain, the Netherlands and the United Kingdom. Across these six major European countries, plant‑based retail sales were worth about €4.7 billion in 2024, with milk and drinks being the largest category and showing continued volume growth. Plant‑based milk and drinks have seen rising sales volumes over the past decade, increasing by more than 150% between 2015 and 2024 across Europe (GFI Europe 2025). Among the various categories, the plant‑based milk alternatives segment remains one of the most established and fastest‑growing parts of the plant‑based market. Globally, plant‐based milk alternatives sales doubled between 2009 and 2015, surpassing $21 billion (Alcorta et al. 2021).

Similarly, plant‑based dairy and meat alternatives have continued to grow, though performance varies across regions. In the USA, total plant‑based retail sales reached approximately $8.1 billion in 2024, with milk alternatives sales around $2.8 billion, while yogurt and creamers alternatives showed modest growth or slight declines (GFI 2024). In Europe, plant‑based milk and drinks remain the largest and fastest‑growing categories, and plant‑based cheese has doubled in value in some markets like Italy between 2022 and 2024 (GFI Europe 2025). The global plant‑based meat alternative market also remains significant, exceeding $6 billion in 2024, although U.S. retail sales have recently stagnated or declined (GFI 2024). New research, R&D investments, inflation, and regulatory changes in the USA may reshape market expectations (Alcorta et al. 2021).

In summary, the rise of the plant‐based sector is driven by lifestyle changes and concerns related to the impacts of animal‐based foods. The market is expected to continue its expansion and exert a growing influence in the near future.

### 2.2 Health Benefits of Plant‐Based Foods

The health effects of plant‐based foods can be evaluated from multiple perspectives. First, consumption of plant‐based food may reduce the risk of chronic diseases. Strict plant‐based diets have been associated with an 8% lower risk of developing coronary heart disease and a 16% lower risk of mortality from coronary heart disease (Kim et al. 2019).

Moreover, phytochemicals and whole plant‐based foods have been shown to reduce levels of low‐density lipoprotein cholesterol (LDL), thereby contributing to plaque formation and increasing cardiovascular disease (CVD) risk. Thus, CVD risk may be reduced, but further research is needed to determine the optimal intake, duration, and composition of such diets (Islam et al. 2021).

A study indicated that high consumption of plant‐based food, combined with low intake of animal‐based foods, significantly reduces the risk of hypertension (Kim et al. 2020). Another study categorized plant‐based diets into three types—overall, healthful, and pro‐vegetarian diets—and including over 2000 participants. While healthy plant‐based foods were linked to a lower risk of hypertension, pro‐vegetarian diets were linked to a higher risk. Overall plant‐based diets were not associated with hypertension risk. These findings highlight the importance of food quality within plant‐based dietary patterns (Kim et al. 2021).

Moreover, strict adherence to plant‐based diets has been associated with reduced risk of prediabetes and insulin resistance (Chen et al. 2020). However, a cohort study emphasized that the quality of plant‐based foods, such as lower sodium content, is associated with a lower risk of type 2 diabetes (Kim and Giovannucci 2022). Plant‐based foods may promote satiety due to their high fiber content, potentially leading to lower energy intake and weight loss (Barnard et al. 2015). On the other hand, one study revealed that plant‐based diets are not consistently associated with obesity, although they may help prevent or manage certain chronic diseases. Additionally, excessive consumption of certain plant foods, especially in the absence of adequate nutrient intake, may have adverse health effects (Magkos et al. 2020).

Maintaining a healthy weight is one of the most effective ways to reduce cancer risk. Combining a plant‐based diet with regular physical activity can facilitate weight loss. This is important because being overweight or obese increases the risk of 12 types of cancer, including colorectal, uterine, kidney, and pancreatic cancers, due to inflammation and hormonal imbalance caused by excess weight (MD Anderson Cancer Center 2020). Plant‐based diets have been shown to reduce inflammation, oxidative stress, and hormonal imbalances. Therefore, they may contribute to the management of cancers such as colorectal, breast, and prostate cancers (Hardt et al. 2022). Additionally, plant‐based foods, such as fruits and vegetables, are characterized by low energy density and high water and fiber content (Hever and Cronise 2017). They contain nutrients that have been clinically studied for their potential effects on depression (La Chance and Ramsey 2018). Plant‐based diets may influence brain function, although current evidence remains inconclusive. The gut microbiome may influence brain function, however, further research is needed to understand how plant‐based diets affect both the microbiome and neurological processes (Medawar et al. 2019). Moreover, current research highlights that although there is no single food that can prevent cognitive decline, leafy green vegetables such as spinach, zucchini, and broccoli may help slow its progression (Harvard Public Health 2024).

Plant‐based foods may support the management of chronic kidney disease in its early stages. Phosphorus, a mineral abundant in many foods, especially animal‐based foods, is removed from the blood by the kidneys. However, in the presence of kidney disease, phosphorus clearance is impaired, leading to its accumulation and potential harm to bones and blood vessels. Plant‐based foods may help reduce phosphorus intake, due to their phytate content, which limits phosphorus absorption. Eating more plant‐based foods and fewer animal‐based foods may help prevent kidney disease or slow its progression if it is already present (National Kidney Foundation 2021).

Research on the relationship between infertility and plant‐based diets remains limited and inconclusive. A study showed that the type of protein may affect semen quality. Higher intake of plant‐based proteins has been associated with lower sperm concentration, though further studies are needed (Abdollahi et al. 2022). Another study suggests that consuming more plant‐based proteins instead of animal proteins may decrease the chance of infertility caused by lack of ovulation (Chavarro et al. 2008). However, further research is still needed to draw definitive conclusions regarding the health effects of plant‐based diets.

In summary, while further research is needed to clarify the health implications of plant‐based diets, current evidence suggests that increased consumption of high‐quality plant‐based foods can support the prevention and management of various chronic diseases. Nevertheless, it is important to consider the nutritional adequacy and diversity of such diets, as excessive or unbalanced intake of plant‐based foods may lead to unintended health outcomes. Therefore, a well‐planned, evidence‐based approach to plant‐based eating is crucial for maximizing health benefits while minimizing potential risks.

### 2.3 Sustainability of Plant‐Based Foods

Food choices affect not only health but also the planet. Every stage of food production emits greenhouse gases (GHG) that increase the Earth's temperature and cause climate change. The global food system accounts for approximately one‐third of all anthropogenic GHG emissions. Animal‐derived foods exert a significantly higher environmental impact compared to plant‐based alternatives (United Nations 2021). Table 1 shows the GHG emissions associated with the production of selected food items.

**TABLE 1:** GHG's per kilogram food (United Nations 2021).

Food (1 kg)	Greenhouse gas emissions (Kg CO_2_e)
Beef	35.5
Lamb	19.9
Cheese	10.8
Fruit	10.4
Milk	9.5
Vegetables	6.8
Fish	6.0
Rice & Grains	4.8
Eggs	4.2
Tofu	2.0
Bread & Pasta	1.3
Legumes	0.9
Nuts	0.3

If current consumption habits continue to 2050, GHG emissions from food production and land use are projected to increase by 80% (Tilman and Clark 2014). Livestock animals like cows and sheep produce methane when they digest their food, thereby contributing significantly to GHG emissions (Abdurehman 2020). The production of animal‐based foods accounts for approximately 15% of all GHG emissions, making it one of the leading contributors to climate change. This figure is comparable to emissions generated by all transportation modes, including vehicles, airplanes, and ships. Moreover, animal agriculture occupies about 70% of all agricultural land, contributing to widespread deforestation, habitat loss, and water pollution (University of Colorado 2022). Transitioning from livestock and animal feed production to plant‐based food systems for direct human consumption can significantly reduce GHG emissions. For example, Denmark reduced agricultural GHG emissions by over 20 mt CO_2_e (86.5%) by reducing meat consumption, decreased reliance on imported animal feed, and increasing plant‐based food consumption (Prag and Henriksen 2020).

In addition, plant‐based foods generally require less land for production compared to animal‐based foods. For example, dairy beef's GHG emissions and land use are 36 and 6 times greater than peas, respectively. Approximately 5% of total calories account for 40% of environmental impacts. A shift to a plant‐based diet can free up agricultural land for biodiversity preservation and the cultivation of food crops for direct human consumption (Poore and Nemecek 2018).

Another significant environmental concern related to food production is water usage. Producing one ton of beef requires approximately 15,400 m^3^ of water, representing the highest water footprint among all food products. Per calorie, this is about 20 times greater than the water footprint of cereals. Additionally, pulse proteins have less water usage than milk and egg proteins, which is almost 1.5 times less (Mekonnen and Hoekstra 2010). Plant‐based food systems offer more efficient resource use, yielding more food with fewer environmental inputs. Compared to animal production, plant cultivation yields more edible calories per unit of resource input such as water, land, and energy (Cassidy 2013). According to Munialo et al. (2022), although plant proteins generally require just 1% of water used for animal protein production, producing 1 kg of isolated soy protein still consumes 38,950 L of water. Therefore, soy protein production may still involve considereble water use despite being more efficient than many animal sources.

Dietary patterns such as veganism and vegetarianism have a significant impact on environmental sustainability. The environmental impact of these diets depends on the specific food choices and the overall nutritional balance. Reducing meat consumption and increasing plant‐based food intake can reduce GHG emissions by up to 70%–80% (Aleksandrowicz et al. 2020). For instance, individuals with high meat consumption produce twice as many GHG emissions as vegans, highlighting the substantial sustainability benefits of reducing animal food intake (Scarborough et al. 2014). Overall, plant‐based foods are considered more sustainable due to their lower use of natural resources and reduced environmental burden.

## 3 Plant‐Based Proteins

Plant‐based proteins are protein‐rich foods derived from plant sources (Tuso et al. 2013). Legumes, cereals, seeds and nuts, soy proteins, lentils, microalgae, plant‐based alternatives such as dairy and meat substitutes, and food waste materials are the main examples of plant‐based proteins.

### 3.1 Legumes and Cereals

Legume proteins are often referred to as “poor man's meat” due to their lower cost compared to animal proteins. Despite being affordable, they are rich in protein. Soybeans have the highest protein content among legumes, while lentils, peas, and chickpeas are also notable sources of plant protein. Legume proteins have good nutritional value and functional properties. They exhibit functional properties such as gelatinization, emulsification, foaming, and solubility during processing and storage. Moreover, legume proteins have been associated with various health benefits. For instance, they may help reduce cholesterol and blood glucose levels as well as reducing the risk of cancers. However, they may also contain allergenic compounds (Mani‐López et al. 2021; Afzaal et al. 2021).

Cereals serve as staple foods in many regions across the world. Wheat, rice, barley, and corn are common cereals. Albumin and globulin are the fundamental proteins for rice, which is a common food worldwide. Albumin contains higher levels of lysine, an essential amino acid, while globulin is richer in sulfur‐containing amino acids. Various techniques have been applied to increase the yield of rice protein isolates, which have relatively low lysine levels. Millet is another cereal widely consumed in developing countries. It is valued for its high nutritional content and abundance of essential amino acids. Cereal proteins are essential components in both industrial food processing and bakery products. In fact, a mixture of legumes and cereals can provide nutritious bakery products by increasing essential amino acids (Langyan et al. 2022).

Pseudo‐cereals are dicotyledonous grains that are gluten‐free. Quinoa, amaranth, and buckwheat are some examples of pseudo‐cereals that have been cultivated for thousands of years worldwide. They possess a nutritional composition comparable to that of conventional cereals. However, differences in starch granules result in distinct functional properties. They also have higher protein content and quality than some cereals, with more essential amino acids such as cysteine and methionine. Nevertheless, further research is required to understand the functional benefits of pseudo‐cereal protein functionality in food production, particularly due to unique protein profiles (Alonso‐Miravalles and O'Mahony 2018). Furthermore, as gluten‐free grains, amaranth and quinoa serve as important alternatives for individuals with celiac disease. Their high lysine content also makes them valuable dietary supplements (Langyan et al. 2022).

### 3.2 Seeds and Nuts

Peanut protein is an essential seed protein for human consumption. It is highly digestible. Peanuts are rich in oil and protein but relatively low in carbohydrates and minerals. The protein content of whole peanuts ranges from 22% to 30%, depending on the shell content (approximately 25%). Peanut flour, protein isolates, and meals can be used to fortify many foods to enhance their sensory qualities (Singh and Singh 1991).

Almonds contain a high protein percentage (21.2%) and are commonly used in various foods. Almonds provide a significant portion of the daily protein requirement for adults. However, they are low in lysine, an essential amino acid, and have moderate protein digestibility. Thus, these proteins are considered to be of low quality (House et al. 2019).

Flax seeds, known as “ *Linum usitatissimum* ”, meaning “very useful,” have been valued for centuries for their nutritional content and health benefits. They are a rich source of plant‐based protein, containing almost 20% high‐quality protein. This protein contains a wide range of essential amino acids. Flaxseed proteins are not only rich in essential amino acids but also offer potential health benefits, such as lowering cholesterol and reducing the risk of cardiovascular disease (Parikh et al. 2019; Singh et al. 2011).

Chia ( *Salvia hispanica* ) is a species in the Lamiaceae family, which includes nearly 900 species. Chia seeds contain approximately 20 proteins with metabolic and lipid‐related functions. Chia protein fractions produce bioactive peptides that exhibit antioxidant and antihypertensive effects. Moreover, albumin and globulin fractions show the highest activity against free radicals, while prolamin and globulin proteins can successfully bind iron, enhancing the antihypertensive and antioxidant effects of the peptides (Grancieri et al. 2019). Additionally, two main types of chia seeds are produced. Black and white chia seeds have approximately equal nutritional values. White chia seeds contain 16.5% protein and 32.4% fiber, while black chia seeds contain 16.9% protein and 32.6% fiber. This difference is attributed to morphology variations (Felemban et al. 2021).

Pumpkin seeds have a high protein content, almost 35%. They contain many amino acids that are beneficial for the diet. In fact, pumpkin seed protein isolates are comparable to soybean protein isolates in terms of bioavailability. They can be used as a nutritious ingredient in food formulations to prevent or address protein malnutrition in vulnerable populations. Additionally, these isolates exhibit potential antioxidant and metal‐binding properties (Dotto et al. 2020). Other plant protein sources also exist, although their utilization remains limited.

### 3.3 Novel Protein Sources

Currently, scientists are working on novel protein sources, such as plant‐based sources, insects, duckweed, microalgae, seaweed, and other marine‐based proteins, in response to growing consumer demand. In addition, utilizing waste streams for protein extraction is another important topic, especially from a sustainability perspective. For example, oil processing generates by‐products containing 15%–50% protein. Oil‐bearing crops such as soybeans, cottonseeds, and peanuts, as well as palm and olive residues, can be utilized for protein isolation (Langyan et al. 2022). Cereal crops and their by‐products such as wheat bran, broken rice, brewer's spent grains, and defatted wheat germ have also been used as protein sources. Pulses also yield by‐products that contain about 25% protein, such as powders, husks, broken seeds, shriveled seeds, and unprocessed seeds. Cereal crops and their by‐products offer high nutritional value and functional potential, a balanced amino acid profile, and various bioactive properties (Amagliani et al. 2017). Therefore, they are considered attractive materials for protein extraction because of their abundance, availability, and amino acid composition.

Moreover, the recombination of plant‐based proteins using certain microorganisms shows potential for use in foods. In this method, microorganisms are genetically enginereed to express plant protein genes to produce proteins that are more digestible, highly nutritious, and less allergenic. Many genes are derived from the genomes of seed storage proteins. Proteins are then produced in media, and biomass is collected from microorganisms to prevent further microbial growth (Karaca et al. 2022).

Microalgae, as another important novel protein source, use photoautotrophic metabolism to convert light and CO_2_ into macromolecules such as carbohydrates and proteins. Moreover, microalgae do not directly compete with conventional crops for arable land and can be used for water treatment and agricultural improvement in arid regions. Microalgae proteins can be processed into various products depending on their protein content and processing level, such as whole‐cell protein and protein concentrates. Whole‐cell proteins consist of intact microalgae cells containing 40%–50% protein. This varies depending on species and growth conditions. Concentrated protein products contain 60%–89% protein and are high‐quality, easily digestible, and rich in essential amino acids. Microalgae proteins can be used in fortified foods, sports drinks, and supplements (Amorim et al. 2021; Soto‐Sierra et al. 2018).

Although many novel protein sources have been proposed by scientists and industry, factors such as sustainability, cost‐effectiveness, supply chain feasibility, safety, sensory properties, and compatibility must be considered when replacing traditional animal‐based proteins (Karaca et al. 2022; Espinosa‐Ramírez et al. 2023). In addition, technical aspects, such as large‐scale protein extraction and purification, must be addressed to ensure efficient and economic use of these novel sources. Furthermore, the presence of anti‐nutritional compounds, contaminants, allergenicity issues, and sensory challenges such as flavor and texture represent major limitations in the industrial application of novel protein sources.

### 3.4 Plant‐Based Analogs

Plant‐based and alternative protein sources have been utilized to develop replacement products such as meat, milk, and dairy products. These products may differ from conventional counterparts, such as cheese, in terms of taste and flavor (Tachie et al. 2023). Various types of meat alternatives are either commercially available or produced at the laboratory scale, including plant‐based options (derived from soy, pea, gluten, etc.), cell‐based, fermentation‐derived, microalgae‐based, and insect‐derived proteins. Cell‐based and fermentation‐derived proteins offer the advantage of forming long fibers, which contribute to a meat‐like texture and may also provide health benefits. However, these protein sources are still under development due to high production costs and associated environmental impacts. In contrast, plant proteins can be directly used to produce meat analogs. Therefore, plant‐based meat alternatives represent a major segment of this rapidly evolving industry (Sha and Xiong 2020). Plant‐based meat substitutes for animal derived ingredients, often with added nutrients to enhance human health. Plant‐based meat products generally contain less protein, fat, and saturated fatty acids compared to animal meat, but more carbohydrates, especially dietary fiber. The main ingredients of plant‐based meat include water (50%–80%), proteins (20%–30%), fats (0%–12%), carbohydrates (6%–15%), and food additives (3%–10%). These components are combined to produce plant‐based meat with a taste and flavor profile similar to that of animal meat. Plant‐based proteins are the key structural components in plant‐based meat. Moreover, protein source significantly influences taste and texture of plant‐based meat. Vegetable oils contribute to the juiciness and flavor of plant‐based meat. Additives are used to improve the texture, flavor, and color of plant‐based meat, which are crucial for consumer acceptance (Wang et al. 2023). Plant‐based proteins serve as an alternative to conventional meat and are primarily derived from soy, gluten, and other legume proteins. A vegetarian diet may be low in certain essential nutrients, such as vitamins B_12_ and D, calcium, zinc, iron, and long‐chained omega‐3 fatty acids. These nutrients can be incorporated into plant‐based meat to improve its nutritional value. However, further research is needed to optimize the processing conditions of plant‐based meat for improved sensory characteristics. For example, simulation techniques can be employed to predict sensory outcomes during production (Hadi and Brightwell 2021).

Plant‐based milk alternatives are water‐soluble extracts derived from legumes, oilseeds, cereals, or pseudo‐cereals, designed to mimic cow's milk. They are typically produced by milling or grinding the raw material, extracting it in water, and then homogenizing the mixture. These products are classified according to the source material: cereals (oats, rice), legumes (chickpeas, soybeans), nuts (almonds, Brazil nuts, cashew nuts, hazelnuts), seeds (sesame, sunflower), and pseudo‐cereals (quinoa) (Silva et al. 2020). While cow's milk contains almost 3.35 g of protein/100 mL, most plant‐based milk alternatives provide lower protein content. Among these, soy milk contains the highest amount, with 3.10 g/100 mL. In comparison, almond milk offers 1.10 g protein, rice milk 0.75 g, and oat milk around 0.30 g/100 mL.

Protein digestibility is another key factor in evaluating nutritional quality. Whey protein isolate has the highest digestibility at 100%, followed by milk protein concentrate at 94%. In contrast, plant‐based proteins generally exhibit lower digestibility values, ranging between 77%–94% (Antunes et al. 2023).

In the production of plant‐based cheese alternatives, plant‐derived coagulants such as artichoke extract and fig tree latex are used in place of animal rennet. The primary ingredients are legumes or nuts, which are combined with commercial fermented yeast and salt. Coconut oil and tapioca flour contribute to the melting and stretching characteristics of the cheese alternatives.

However, plant‐based cheese alternatives face challenges in replicating the meltability, stretchiness, and texture of traditional dairy cheese. Plant proteins do not share the same molecular and functional characteristics as milk casein. As a result, plant‐based alternatives are generally more successful when aiming for soft and creamy textures, such as those found in feta, ricotta, or cottage cheese (Mefleh et al. 2021).

Plant‐based yogurt alternatives are typically produced by fermenting different plant materials, including cereals, pseudo‐cereals, and legumes. However, without the addition of stabilizing ingredients, the protein structure in these products does not fully resemble that of traditional yogurt. Because plant proteins generally contain less protein and differ in structure from animal proteins, they coagulate differently and often fail to form a stable gel matrix. As a result, phase separation or emulsion breakdown may occur after fermentation. This issue can be addressed by incorporating additives such as protein extracts, inulin, thickeners, and emulsifiers. They are usually lower in protein than dairy yogurt, but they can have higher protein digestibility and bioavailability due to fermentation and enzymatic treatments (Montemurro et al. 2021).

Despite the growing interest in plant‐based analogs of animal products, several critical issues remain during their production and consumption. These include the need for more intensive processing compared to animal products, high sodium content, undesirable off‐flavors, low textural quality, and the absence of specific meat‐related compounds, vitamins, and volatiles. These limitations represent key limitations of current analog products. Further research is essential to address and overcome these challenges.

## 4 Comparison of Animal Proteins and Plant‐Based Proteins

Although there are differences between plant‐based and animal proteins, major health authorities recommend including both in a balanced diet, albeit in different proportions. For example, Harvard Public Health School suggests that healthy proteins should occupy one quarter of the plate. It recommends foods such as fish, poultry, beans, and nuts, especially when paired with salads and vegetables, while advising that processed meats like sausage be limited (Harvard Public Health 2022). Similarly, Canada's Food Guide advises the daily consumption of plant‐based protein sources, noting their higher fiber content and lower levels of saturated fat compared to animal‐based proteins (Health Canada 2022). In the United Kingdom, dietary guidance emphasizes a greater intake of beans, lentils, peas, and other plant‐based proteins over animal‐based options, citing health and environmental benefits. However, this does not imply that all animal foods should be avoided; rather, both types are important for health (British Nutrition Foundation 2022). In short, a flexible dietary pattern that incorporates both plant and animal protein sources is recommended. Table 2 shows the key differences between these proteins for several aspects.

**TABLE 2:** Comparison of animal and plant‐based proteins.

Animal proteins	Plant‐based proteins	References
Account for 20% of total protein production worldwide.	Account for 80% of total protein production worldwide.	(Langyan et al. 2022)
Meats, milks, fishes, eggs, poultry, yogurts are some sources of animal proteins	Cereals, legumes, seeds, nuts, plant‐based alternatives are some sources of plant‐based proteins	
Provide 43% of the global protein supply in the world but Most people take protein from animal products every day.	Provide 57% of the global protein supply.	
Supply essential amino acid amounts with proteins	Inefficient to provide essential amino acids requirements generally	(Ewy et al. 2022)
Too much consumption causes overweight and chronic diseases like type 2 diabetes	Feels good and helps your gut and the microbiota in it stay healthy due to fiber content	
High digestibility property	Lower digestibility property	
Higher protein content and bioavailability	Lower bioavailability (70%–99% protein bioavailability)	(Wang et al. 2022)
Only lower intakes decrease cardiovascular disease risk	Higher intakes provide cardiovascular health	
More animal protein consumption is related to prostate cancer risk	Increased plant‐based protein consumption decreases prostate cancer risk	(Shin et al. 2019)
For every 10% increase in animal protein intake, the risk of death from heart disease is 8% higher	For every 3% increase in plant protein intake, the risk of death from any cause is 10% lower	(Petersen et al. 2017)
For every 5% increase in animal protein intake, the risk of type‐2 diabetes is 8% higher	Not associated with type‐2 diabetes risk factor	
Higher muscle protein synthesis especially whey protein	Lower muscle protein synthesis ratio but they can be suitable with fortification	(van Vliet et al. 2015)
Increase saturated fatty acids intake	Increase fiber intake and dietary fat quality	(Päivärinta et al. 2020)
For every 100 g of protein: beef—35.5, cheese—10.8, milk—9.5, eggs—4.2 (kg CO_2_e GHG emissions)	For every 100 g of protein: fruit—10.4, vegetables‐ 6.8, grains—4.8, legumes—0.9, nuts—0.3 (kg CO_2_e GHG emissions)	(United Nations 2021)
Higher GHG emissions due to production processes	Lower GHG emissions between 75%–95% than animal proteins	(Kumar et al. 2022)
80%–95% more water usage and requires more energy	More than 99% less land usage	
More opportunity food losses	More efficient opportunity food losses	(Shepon et al. 2018)
Have contamination risks like *Salmonella, Listeria* etc.	Have more contamination risk especially plant‐based alternatives from ingredients	(Tachie et al. 2023)
Higher functionality such as foaming, gelling, textural and sensory properties	Low functionality due to hydrophobicity, aggregation, and inflexibility	(Day et al. 2022)
Higher in calories and minerals Na, Ca, Zn, PO_4_ and vitamin B_12_	Lower in calories	
Proteins with less than 54% similarity to human homologs are generally considered allergenic, whereas those with more than 65% similarity are rarely associated with allergenicity	Among the 467 identified plant allergens, 436 belong to specific protein families	(Breiteneder 2008; Costa et al. 2022)
Milk, eggs, and fish are common allergens derived from animal protein sources	Wheat, soy, lupin, pea, peanut, and hazelnut are known allergens among alternative plant protein sources	(U.S. Food and Drug Administration 2024; Jappe 2023)

## 5 Consumer Perspectives

The demand for alternative proteins is increasing globally, driven by health and environmental concerns. However, factors such as food neophobia, cultural habits, and unfavorable sensory properties negatively affect the adoption of alternative proteins. According to Malek and Umberger (2023), four in ten Australians do not consider the consumption of alternative proteins important, while three in ten Australians are actively trying to increase their intake. The main reasons consumers avoid alternative proteins are a lack of interest (66%) and dissatisfaction with their sensory properties (37%). Consumers' familiarity with the sensory properties of animal proteins, combined with their taste and mouthfeel expectations shaped by these products, limits their acceptance of plant‐based proteins, as these expectations are often not fully met (Karabulut et al. 2024). Improvements in taste, price, health benefits, and nutritional value are necessary to encourage consumers to shift toward alternative proteins. The bitter and acidic taste of plant‐based proteins is often perceived unfavorably by consumers; therefore, masking agents are used to enhance the taste of plant‐based food alternatives (Aimutis 2022). However, inadequate flavor masking remains a major issue that negatively affects consumer perception. The tendency to consume alternative proteins varies across countries and between different types of proteins. A study conducted in the United Kingdom, Spain, Brazil, and the Dominican Republic found that the willingness to give up traditional meat was 200% higher in the economically developed United Kingdom and Spain compared to Brazil and the Dominican Republic. Consumers in all four countries prioritize food safety, taste, affordability, nutritional content, and environmental benefits when choosing alternative proteins, similar to their preferences for other foods. Although animal meat consumption is lower in the Dominican Republic than other countries, it exhibits the most conservative attitudes toward alternative proteins due to cultural factors (Gomez‐Luciano et al. 2019).

In EU countries, legumes are the most preferred source among alternative proteins. Few consumers show interest in cell‐cultured meat, and a significant portion holds negative attitudes toward it, perceiving it as unnatural. In addition, insects, unlike in parts of Asia where they have been consumed for centuries, are not well received by consumers in EU countries and are considered among the least accepted alternative sources (Faber et al. 2023). Efforts are being made to change the perception that cultured meat is artificial, and if their prices approach those of conventional meat, EU consumers may respond more favorably. EU consumers believe that insects can be consumed due to their sustainability, as long as they are not visibly recognizable during consumption. All barriers that hinder the consumption of alternative proteins such as cultural differences and sensory properties also impede the adoption of insect‐based proteins (Siddiqui et al. 2022). Among alternative proteins, one of the product groups that does not have a large market size is algae. A study conducted in the United Kingdom indicates that increasing awareness of algae as a novel protein source and informing consumers about its properties can increase their willingness to purchase it (Mellor et al. 2022). Plant‐based proteins are the most preferred alternatives across different regions because they have been present in the market longer than other meat substitutes (Gomez‐Luciano et al. 2019). Although consumers often regard plant‐based foods as healthy, environmentally friendly, and ethical, they are perceived as lacking value within the production chain. This perception within the supply chain is largely due to insufficient information and a lack of clear regulations (Blanco‐Gutiérrez et al. 2020). In the United States, 25% of consumers prefer plant‐based meat when given a choice between traditional and plant‐based options. Price remains the key determinant in protein choice, and American consumers who prefer plant‐based proteins are typically affluent, young, reside in the Western United States, and identify as Democrats (Tonsor et al. 2023). Conversely, in ten EU countries, key barriers to adopting alternative proteins include cost, availability of foods, and taste. To persuade the 37% of the respondents unwilling to alter their diets, a communication strategy emphasizing their potential individual contribution to sustainability is necessary. Furthermore, education level and place of residence influence the extent to which individuals experience barriers to dietary change, even at relatively low levels (Perez‐Cueto et al. 2022). While education positively influences the selection of plant proteins, affordability and acceptable sensory properties remain critical factors in consumer preference. The long‐standing availability of plant proteins compared to other alternatives may position them as the primary choice when transitioning away from animal proteins. Given the extended time required for developing new alternative proteins and the escalating impact of the global climate crisis, it is inevitable that plant proteins will become the leading source of sustainable protein. As the significance of plant‐based proteins continues to grow, consumer perception has become increasingly important. Table 3 presents consumer studies related to plant‐based proteins and products incorporating these protein sources.

**TABLE 3:** Consumer perception on plant‐based proteins and sources.

Objective	Location	Results	References
To explore consumer perceptions regarding the sustainability of dairy products and plant‐based dairy alternatives (PBDA).	USA	The most essential points for dairy sustainability are lowering GHG emissions, avoiding preservatives, animal welfare, and minimizing ingredients. Sustainability is essential but consumers do not perceive a clear relationship between dairy products and PBDA. Consumers may misjudge that PBDA are more sustainable than dairy products because of a lack of knowledge and reliable information.	Schiano et al. (2020)
To identify effective communication strategies on hybrid meats for European policymakers and producers.	United Kingdom, Denmark, and Spain	Hybrid meat products face difficulty in gaining consumer acceptance, as they are more expensive than conventional meat products. Meat‐only burgers are preferred by consumers in the three countries, while hybrid and plant‐based burgers are disliked because of unfamiliarity. Sensory and health information such as “reduced fat” have a positive effect on consumer acceptance for plant‐based food.	Asioli et al. (2022)
To examine the outlook of healthcare professionals and develop accurate guidance strategies for consumers.	USA	Health professionals who are not related to dietetics have a significant lack of knowledge about the nutritional adequacy of plant‐based dairy alternatives. Many health professionals think that consumers cannot understand the nutritional differences between dairy products and plant‐based dairy alternatives.	Clark et al. (2022)
To evaluate consumer opinions on conventional meat, cultured meat and plant‐based meat alternatives.	Australia	Plant‐based meats are the preferred option to replace meat for consumers concerned about ecological and animal welfare issues. Consumers believe that the biggest barrier to plant‐based meats is taste. Consumers' perception is that plant‐based meats are almost as safe as conventional meats.	De Oliveira Padilha et al. (2022)
To assess consumer demand for plant‐based and animal‐based proteins.	USA	Some consumers prefer both beef and plant‐based proteins on the same day, as they do not seem as alternatives to each other. Animal welfare, health and environmental concerns are the main reasons for preferring plant‐based proteins. Beef is preferred for its protein and iron content, while plant‐based protein are chosen for their dietary fiber and lower fat content.	Taylor et al. (2022)
To measure the balance between plant and animal proteins in food services offered to consumers.	Australia and New Zealand	Rearranging menus is one of the most effective ways to increase plant‐based protein consumption. Recipe modifications and menu labeling do not negatively affect consumer preferences for plant‐based proteins. A plant‐forward dietary style highlights plant‐based foods without excluding other food types.	Stiles et al. (2022)
To study consumer perceptions of the environmental impact of food products.	Switzerland	Consumers perceive the environmental impacts of soy‐based meat alternatives as similar to those of conventional meat. New protein products may not reduce the demand for animal proteins, but they can expand the overall market for protein‐rich foods. Awareness of the environmental impact of foods influences consumer behavior.	Siegrist and Hartmann (2019)
To investigate trends in plant‐based alternatives and related consumer perceptions.	United Kingdom	Plant‐based food alternatives make it easier for meat consumers to shift their diets due to environmental and health concerns. Proper regulations can help prevent future public health concerns. Women tend to feel more responsible and consume more plant‐based foods than men.	Alae‐Carew et al. (2022)
To examine the perceptions of Australian consumers and nutrition professionals regarding plant‐based proteins.	Australia	Plant‐based alternatives are not yet perceived as a social norm among consumers. Plant‐based burgers and sausages are appealing to consumers, whereas sea food alternatives are generally less favored. Nutritional professionals express concerns about nutritional adequacy, but they believe that whole foods can meet nutritional needs.	Estell et al. (2021)
To describe consumer insights into alternative meat products.	Germany	More than 50% of consumers occasionally consume meat alternatives due to environmental concerns. Hybrid meats, which include both animal meats and plant‐based proteins, are the most preferred alternative. Food neophobia is a significant barrier to the consumption of alternative meat products.	Profeta et al. (2021)
To compare consumer perceptions of different protein sources.	France	The composition and formulation of plant‐based meat alternatives significantly affect consumer liking. Educating consumers about the sustainability and health benefits of plant‐based foods promotes their consumption.	Cordelle et al. (2022)
To compare consumer perceptions of plant‐based and cultured meats.	Belgium	Cost, authenticity, wellness, and consumer confidence are the main barriers to the adoption of alternative meats. Consumer satisfaction with alternatives increased from 44% to 51% between 2019 and 2020. Women are more likely to consume plant‐based meat, whereas men tend to prefer cultured meat.	Bryant and Sanctorum (2021)
To measure consumer attitudes toward plant‐based egg alternatives.	United Kingdom and Italy	Egg‐shaped plant‐based alternatives are most commonly preferred in the UK. Egg powder and liquid plant‐based eggs are most favored by Italian consumers. Price, sustainability, practicality and taste are the key factors influencing the preference for plant‐based egg alternatives.	Rondoni et al. (2021)
To compare consumers perceptions of plant‐based milk alternatives and dairy milk in coffee.	Canada	Traditional milk is more preferred than oat or almond milk alternatives. Plant‐based consumers can distinguish almond and soymilk alternatives when added to coffee, whereas dairy consumers typically cannot.	Gorman et al. (2021)
To evaluate the sensory attributes of plant‐based chicken alternatives.	Canada	Consumers can differentiate plant‐based chicken alternatives based on taste and texture. Consumers emphasized that plant‐based chicken alternatives are not ideal for flavorful meals, but they are willing to consume them for sustainability reasons.	Ettinger et al. (2022)
To evaluate the impact of packaging color and design on consumer evaluations of plant‐based meat alternatives.	Canada	Plant‐based meat alternatives are perceived as more eco‐friendly and healthier when presented with neutral packaging colors. Red packaging is associated with the term “meat”, even when the product is a plant‐based alternative.	Sucapane et al. (2021)
To evaluate consumer perceptions of alternative protein sources (plant‐based, lab‐grown and edible insect) compared to animal proteins.	United Kingdom	The primary reason for preferring plant‐based proteins is their perceived health benefits. Lab‐grown meat may serve as an alternative for meat consumers, while plant‐based meat alternatives can appeal to both meat eaters and vegans.	Circus and Robison (2018)
To identify key factors influencing consumer choices related to protein sources.	USA	The source of protein is not too important for consumers. Protein claims on product labels are positively perceived by consumers. Food neophobia can influence the overall evaluation of new protein sources.	Antoniak et al. (2022)
To gain insight into consumer perceptions of conventional versus alternative proteins.	Portugal	There is no single approach to promoting alternative proteins that works for all consumers. Hedonically motivated consumers tend to dislike meat alternatives, whereas ethically driven consumers prioritize plant‐based protein alternatives.	Possidonio et al. (2021)
To evaluate consumer behavior regarding healthiness and environmental friendliness.	Switzerland	People can distinguish between meat, milk, and plant‐based alternatives based on whether they perceive them as ecofriendly or healthy. Among these options, plant‐based proteins are often perceived as the healthiest and most environmentally friendly products.	Lazzarini et al. (2016)
To measure consumer motivations behind the preference for meat and meat alternatives.	Spain	The method of production is a major reason for the rejection of plant‐based meat alternatives. Consumers who prioritize natural production tend to prefer plant‐based meat alternatives over cultured meats.	Escribano et al. (2021)

Studies from various regions show that plant‐based food alternatives require greater recognition and awareness regarding their nutritional composition. In particular, consumers in European countries are shifting toward plant‐based proteins due to health and environmental concerns. However, factors such as cost, sensory properties including taste and appearance, and food neophobia affect consumer preferences. These factors may serve as barriers to food choice and can be just as effective as environmental and health concerns. In the United States, plant‐based meat alternatives are not commonly associated with animal meat products. Additionally, price and protein content labeling play an important role in the selection of protein alternatives, whereas the protein source itself is considered less important. However, consumers have higher levels of awareness in Canada. On the other hand, Australian consumers struggle to align plant‐based food alternatives with prevailing social norms, although they generally do not react negatively to them. Notably, in Switzerland, where education levels are high, plant proteins are the primary alternative protein, as their impact on the environment and health is known. Most studies do not consider the impact of allergens on consumer perception. It is estimated that around 10% of adults and 8% of children have food allergies, and these percentages are increasing over time (Tanno and Demoly 2022). The gap in the literature is expected to be addressed by future studies, particularly as the consumption of allergenic ingredients such as soy and pea protein, commonly used in plant‐based food alternatives, continues to rise. In summary, increasing awareness of the health and environmental advantages of plant‐based foods, along with improvements in sensory attributes, accessibility, and cultural integration, will enhance consumer preference and acceptance.

## 6 Challenges of Plant‐Based Foods and Proteins

Several challenges hinder the development of plant‐based proteins, including consumer‐related, health, and sustainability aspects. Although plant‐based proteins offer health and sustainability benefits, they also face challenges. Many consumers may not fully recognize the importance of plant‐based foods for health and environmental sustainability. Additionally, consumers may hold negative perceptions about plant‐based proteins, such as perceiving them as less flavorful and more expensive (Aschemann‐Witzel et al. 2021). Moreover, food neophobia may reduce plant‐based food consumption (Alcorta et al. 2021). Food neophobia refers to the reluctance to try new or unfamiliar foods, particularly among children aged 2‐6 years, and is influenced by genetic, psychological, and environmental factors (Dovey et al. 2008). New foods, technologies, brands, or products from unfamiliar regions can trigger food neophobia. To minimize it, increased exposure through travel, cultural connections, and urban environments is recommended. Social influences can also encourage individuals to be more open to trying new foods (Siddiqui et al. 2021).

The sensory attributes of plant‐based foods are often perceived as inferior to those of animal‐based products in terms of appearance, texture, and taste. Therefore, plant‐based products should be formulated to align with consumer expectations. Although consumers acknowledge the advantages of plant‐based foods, taste remains a critical factor (Sloan 2021). According to the Eurobarometer 2022 Report, taste is the second most important factor influencing food choices (European Commission 2023). Although demand for plant‐based products is increasing, European consumers face an important barrier to purchasing them. Plant‐based protein alternatives tend to be more expensive than animal‐based counterparts (ProVeg International 2020). Among the key factors influencing food preferences, cost (54%) is reported as the most frequently cited by participants (European Commission 2023).

Moreover, animal foods, such as meat, have social norms and rituals within societies. Therefore, it may be very difficult for some societies to completely remove animal foods from their diet and accept plant‐based foods. For instance, many cultures and events regard meat as a sign of prestige, manliness, authority, and heritage. Thus, plant‐based foods may have different meanings and symbols in different communities (Tyndall et al. 2024).

The Court of Justice of the European Union decided that terms such as “milk,” “cream,” “butter,” “cheese,” or “yogurt” can only be used for animal‐derived products. Thus, plant‐based products cannot use these terms to market themselves (Court of Justice of the European Union 2017). In addition, France has banned the use of terms like “sausage” and “bacon” for plant‐based foods (Conseil d'État 2021). This limitation affects not only the use of names but also the marketing of the products. For example, since 2022, the sale of plant‐based cheese in the markets has been prohibited in Türkiye due to the use of the term “cheese” (The Vegan Association of Turkey 2022). However, updated 2025 food labelling guidance now allows these products to be marketed and sold if appropriately labelled without terms associated with animal products (Republic of Turkey Ministry of Agriculture and Forestry 2026). All these limitations are due to the lack of regulations on plant‐based food alternatives. Thus, the U.S. Food and Drug Administration (FDA) has worked on “Labeling of Plant‐Based Milk Alternatives” (U.S. Food and Drug Administration 2023). In addition, the ISO 8700:2025 Plant‐based Foods document, which defines terms and properties of plant‐based foods, was published in 2025 (ISO 2025). However, regulations and legislation focusing on these alternative sources are still needed.

On the other hand, plant‐based foods and proteins can provide sustainability benefits, such as reducing CO_2_ emissions, water and land use, and helping preserve biodiversity (Torkington 2022). However, there are also some sustainability challenges associated with plant‐based foods. For example, it was reported that plant‐based cheese samples lack life cycle assessments (LCAs) for production processes (Grossmann and McClements 2021). Additionally, public awareness of the negative environmental impacts of animal‐based production and consumption is limited. For instance, most people in Western countries are reluctant to reduce or stop meat consumption for environmental reasons. Even for vegans and vegetarians, environmental concerns often follow the primary motivation for their diet, which is animal welfare (Sanchez‐Sabate and Sabate 2019).

In summary, there are still some challenges to be solved, especially from the consumer perception point of view. Resistance to changing dietary habits, dissatisfaction with the textural and flavor characteristics of novel foods, and concerns about the safety of these products are the main problems. From a production and processing perspective, heavy processing and challenges in scaling up the extraction and purification of plant‐based proteins are critical issues that require further attention. In terms of consumer health, excessive consumption of plant‐based products may lead to challenges such as the presence of anti‐nutritional factors and allergens, and high sodium levels. The lack of uniformity in legislation and definitions regarding plant‐based products is also an important issue that needs further efforts and collaboration among policymakers, scientists, and industrialists.

## 7 Conclusion

Protein is essential at all life stages, people need protein to produce energy, maintain and repair cells and tissues, build strength, and support various metabolic functions of the human body. A lack of protein leads to various health problems, and a significant portion of the global population is already affected by this issue. The world's population is increasing significantly, and the requirement for food will increase by up to 70% by 2050.

The plant‐based foods market in Europe was valued at approximately €9 billion in 2024. The plant‐based meat, milk, and dairy markets also experienced significant growth in both the EU and USA markets. Several factors have contributed to these developments. Firstly, plant‐based foods have significant health benefits. They may reduce chronic diseases such as type‐2 diabetes, high cholesterol, and hypertension. They help regulate calorie intake and body weight. In addition, plant‐based diets have been linked to the prevention and management of certain types of cancer, cardiovascular diseases, and chronic kidney disease. However, the effects of plant‐based diets on mental health remain unclear and require further research. Moreover, plant‐based foods contribute to sustainability. Unlike animal‐based foods, plant‐based alternatives can reduce GHG emissions, land and water use, and deforestation.

Although plant‐based foods have many advantages, they also face several challenges. From a consumer perspective, the benefits of plant‐based foods are not widely recognized, and negative perceptions related to taste, price, and food neophobia are common. In terms of regulations, some terms are restricted, such as “plant‐based milk” in the EU and “plant‐based bacon” in France. However, the FDA continues to develop on regulations for plant‐based foods. Moreover, plant‐based proteins are often insufficient in essential amino acids and exhibit relatively lower bioavailability and digestibility. From a sustainability perspective, plant‐based proteins generate lower GHG emissions and require less land and water compared to animal proteins. However, they tend to exhibit relatively lower functionality, such as gelling, emulsifying, and foaming. Thus, extra effort is needed to create alternatives to animal products. While many health authorities recommend consuming both animal and plant‐based proteins, plant‐based sources are often preferred due to health and environmental concerns related to animal products.

Consumer perspectives on plant‐based proteins and alternatives vary across countries and demographic groups. Despite the growing popularity of plant‐based products, many consumers lack awareness and understanding of them. Consumption behaviors and taboos surrounding plant‐based proteins can be barriers to the increased consumption and large‐scale production of these products. Additionally, issues such as price, taste, and accessibility play important roles in consumer preferences.

In summary, plant‐based foods are emerging as key components of future food systems. Arranging necessary policies and legal regulations, researching the elimination of taste and flavor problems, and improving the digestibility and techno‐functional properties of plant proteins, along with increasing awareness of the effects of plant‐based foods on environmental and sustainability issues, will lead to better consumer preferences.

## Author Contributions

**Esra Capanoglu:** conceptualization, writing – review and editing, project administration, investigation. **Batuhan Inanlar:** investigation, writing – original draft.

## Funding

This research did not receive any specific grant from funding agencies in the public, commercial, or not‐for‐profit sectors.

## Conflicts of Interest

The authors declare no conflicts of interest.

## Data Availability

The data that support the findings of this study are available from the corresponding author upon reasonable request.
